# Two-Dimensional Radiographic Diagnosis of Maxillary Canine Impactions

**DOI:** 10.3390/dj12110360

**Published:** 2024-11-13

**Authors:** Alin M. Iacob, Matías Ferrán Escobedo Martínez, Sonsoles Olay García, Sonsoles Junquera Olay, Luis Manuel Junquera Gutiérrez

**Affiliations:** 1 Department of Integrated Adult Dentistry, School of Dentistry, University of Oviedo, C/. Catedrático Serrano s/n., 33006 Oviedo, Spain; uo272130@uniovi.es (A.M.I.); olaymaria@uniovi.es (S.O.G.); 2 Department of Radiology, Hospital Universitario Central de Asturias, 33004 Oviedo, Spain; sonsoles.junquera@sespa.es; 3 Department of Oral and Maxillofacial Surgery, School of Dentistry, University of Oviedo, C/. Catedrático Serrano s/n., 33006 Oviedo, Spain; junquera@uniovi.es

**Keywords:** maxillary canine, impaction, impacted canine, treatment, diagnostic methods

## Abstract

**Background:** The primary aim of this study was to evaluate, using conventional radiographic imaging, the height, angulation, and mesiodistal position of impacted maxillary canines to determine if these variables are significantly associated with the palatal or buccal positioning of the tooth. **Methods:** A retrospective analysis was conducted on all patients diagnosed and treated for at least one impacted maxillary canine over a 4-year period in the Principality of Asturias. The final sample included 159 impacted canines. The variables analyzed were age, gender, associated pathology, location, angulation, height, mesiodistal position, buccopalatal position, and treatment method. Follow-up periods ranged from 12 to 50 months. **Results:** The mean age of the patients was 21 years (range 10–41 years), with most cases occurring in patients aged 14 to 30 years. Females accounted for 66.1% of the sample, with a female-to-male ratio of 1.95:1. The type of treatment (extraction/traction, surgical–orthodontic approach) was significantly associated with patient age. Additionally, the proximity of the impacted canine crown to the palatal or buccal cortices was significantly related to the treatment choice. However, no significant relationship was observed between the palatal/buccal position of the canine crown and its height or angulation. **Conclusions:** In the present work, the upper canines included by the palatal did not have a greater height or angulation than the canines included by the vestibule.

## 1. Introduction

Impacted teeth are those that, whether partially or fully developed, remain embedded within the jawbone beyond the typical eruption period. Dental impactions are a common condition encountered frequently in oral and maxillofacial surgery [1,2].

Unlike third molars, the aesthetic and functional importance that canines play strongly conditions their treatment. The goal of this project was to promote the eruption of the impacted tooth so that it could fulfill its crucial role in guiding occlusion [3,4,5,6,7,8,9,10,11].

The mineralization of the crown of the upper canines begins between 4 and 12 months of age and is completed by 6 to 7 years. The root is fully formed around the age of 13.6 years of age. The tooth emerges when about three-quarters of its root are developed, with the lateral incisor guiding the canine’s eruption. The agenesis of lateral incisors is linked to a higher incidence of canine impactions [1,4,5,12]. In the final year before eruption, the dental follicle appears to play a critical role in the eruption process.

Upper canine eruption varies by gender, typically at 12 years for females and 13 for males. Canines remaining unerupted after the age of 14 years are often pathological. Eruption is usually symmetrical, but up to 10% may show a one-year asymmetry.

The prevalence of bone impaction for these teeth ranges from 0.92% to 2.1%, primarily affecting females. Cone beam computed tomography (CBCT) studies show that the canine crown is usually in the palatal position (49.2%), followed by the labial (37.1%) and mid-alveolar (16.3%) positions [13].

The lack of canine eruption has multifactorial causes, including insufficient arch space, agenesis of lateral incisors, ankylosis, delayed exfoliation of deciduous canines, malposition, hereditary factors, trauma, palatal clefts, cysts, tumors, root alterations, and idiopathic cases [14,15].

Clinically, the lack of eruption can lead to aesthetic and functional issues, as well as root resorption of adjacent permanent teeth, with literature indicating a 38–66.7% risk for lateral incisors [13,14,15].

The diagnosis of this pathology is based on adequate oral inspection and palpation, and the study is completed by intra- and extraoral, two-dimensional radiographic methods and three-dimensional methods [5].

However, a major limitation of these basic studies is that they fail to clearly identify the condition of the lateral incisor root in at least 97% of cases. Consequently, three-dimensional imaging with computed tomography is considered the gold standard for an accurate diagnosis [8]. At present, the CBCT, as set out in the DIMITRA project (dentomaxilofacial pediatric imaging investigation toward low-dose radiation)*,* can be considered a justified technique in children for the diagnosis and treatment planning of impacted teeth and root resorption. In some studies, more than 25% of the treatment plans initially proposed with two-dimensional studies were modified when consulting CBCT images [16].

The best way to treat the maxillary impaction of canines is to proceed with the extraction of decidual canines between nine and eleven years of age. To do this, an early diagnosis of the problem is essential [5]. In patients under 14 years of age, Sinha et al. reported normalizations in the eruption of the canine when the ectopic impaction of its crown was located distal to the middle root portion of the lateral incisor, lowering this percentage to 64% when it was located mesially [17]. Above the age of 14 years, when dental impaction is already established, treatment options include three major possibilities: therapeutic abstention, extraction of impaction and surgical–orthodontic treatment to pilot the tooth to its natural location. Of all of them, the latter is the one that is emerging as the first option, although its application is not always feasible [5].

The literature indicates that the surgical–orthodontic treatment of canines impacted in the palatal position is generally more challenging than for those located closer to the buccal cortical bone [3,7,8,10]. Now, the greater thickness of the mucosa and palatal cortical bone, as well as the lack of space in the arch, much more frequent in palatal impactions, are postulated as justifying factors for this disparate difficulty. However, it is important to consider that other factors not investigated to date could play a significant role in this variability in observed clinical behavior. These factors include the height of the included canine, the angulation of the tooth relative to adjacent structures, and potentially other anatomical and physiological parameters that have not yet been exhaustively studied. These aspects could influence the complexity of the necessary surgical–orthodontic procedure and the prognosis of treatment, underscoring the need for further research to fully understand the variables that affect the success of the management of the included canines [5].

In line with the above, there were three clinical questions of interest in this work. What are the epidemiological characteristics of impacted upper canines diagnosed and treated in our environment? What variables determine a greater tendency to extract these impacted teeth, compared to more conservative treatments? Do the height, angulation, and mesio-distal position of impacted canines have a significant relationship with the palatal or vestibular location of the tooth recognizable in the orthopantomography?

Our working hypothesis (H0) remains: “The upper canines included by the palatal area have a greater height or angulation than the canines included by the vestibular area”.

## 2. Materials and Methods

### 2.1. Subjects of the Study

**Figure 1:**
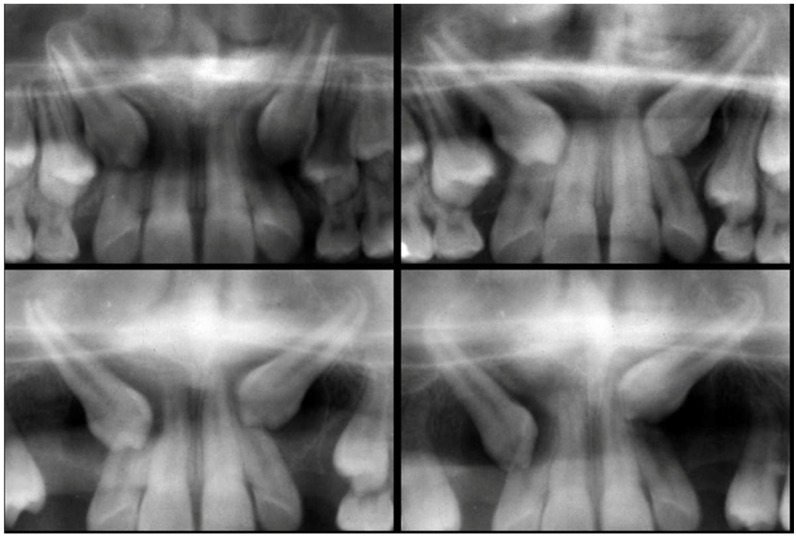
Orthopantomography of the canines included bilaterally. Relationship of its crown to the root of the lateral, as the root formation of the canines is completed.

**Figure 2:**
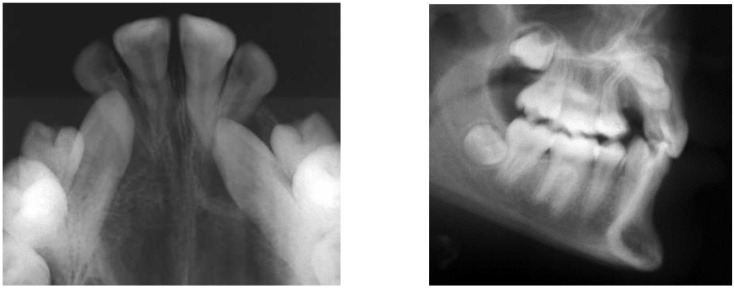
Occlusal X-ray and lateral teleradiograph corresponding to the patient in Figure 1.

**Figure 3:**
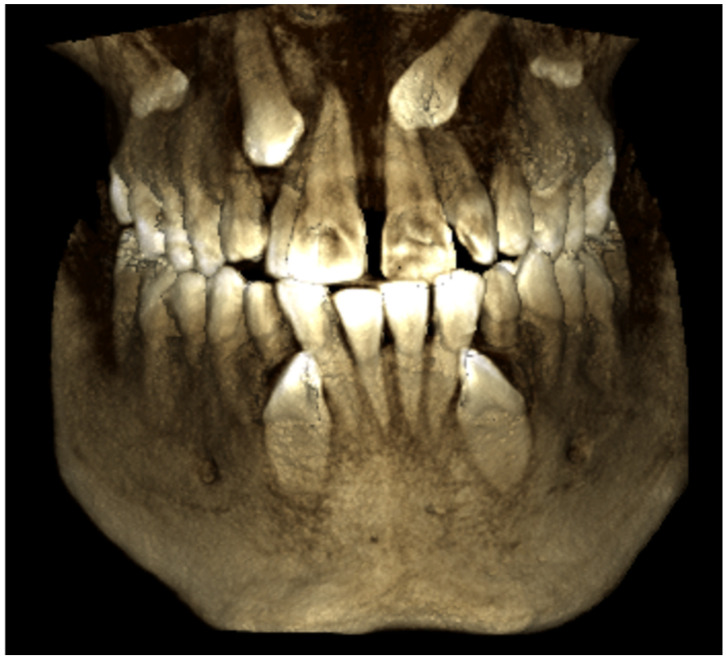
CBCT showing impactions of the four definitive canines and their relationship to adjacent teeth.

A retrospective study was carried out that included all patients diagnosed and treated with at least one canine included, within a period of 4 years (2019–2022) in the Principality of Asturias, Spain. These patients were treated in the private clinics of different orthodontists and operated on by a single surgeon at a private center. All patients underwent a medical history, stomatognathic examination, conventional radiographic studies (orthopantomography, occlusal, and/or lateral teleradiograph) (Figure 1, Figure 2 and Figure 3), impression taking, and dental casts, impression taking, and dental casts. The final therapeutic plan was agreed upon by the different specialists involved in the patient’s treatment. The follow-up of the cases covered a period between 12 and 50 months. All patients provided signed consent, allowing the use of their images for research and educational purposes.

The sample size of 136 patients included in this study was determined based on the specific criteria established for patient selection. Although there were many patients with impacted canines in our center, we applied strict inclusion criteria to ensure the quality and reliability of the data. Specifically, we focused on patients who had clear radiographic images and well-documented diagnoses. After filtering through the initial cohort, we identified 136 patients who met these criteria, accounting for a total of 159 impacted canines. This rigorous selection process allowed us to focus on cases with comprehensive diagnostic information, thereby enhancing the validity of our findings while ensuring a manageable sample size for analysis. The study was conducted in accordance with the Declaration of Helsinki, and approved by the Ethics Committee of Principado of Asturias, Spain (protocol code CEIm number 2018.528 and date of approval 20 December 2018).

### 2.2. Data Collection

A singled-out database was developed using *Microsoft’s Access* program. In it, and for each dental impaction, the following variables were collected: age, gender, associated pathology, location, angulation, height, mesio-distal position, vestibulo-palatal position, and treatment applied.

**Figure 4:**
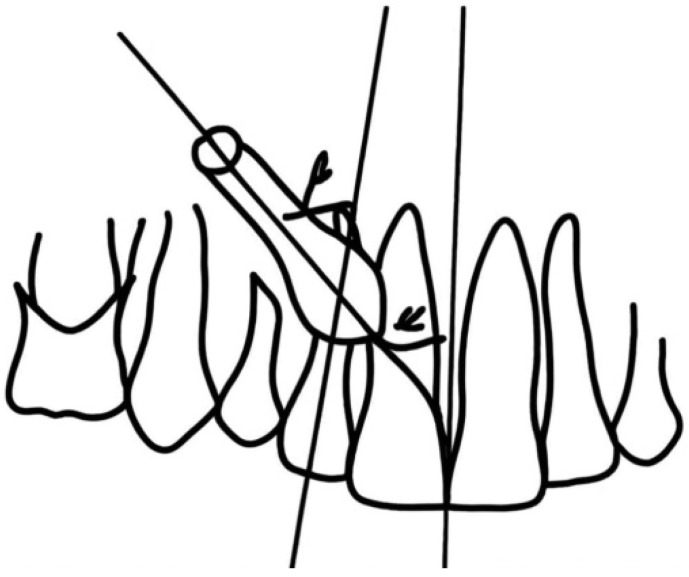
Angulation of the impaction.

The subjects were divided into three subgroups according to age: children (10–14 years), young (15–30 years) and adults (over 30 years). For the location variable, a distinction was made between the maxillary or mandibular location of the impaction, and the right or left location of the impaction. Two procedures were used to study angulation [18]: (a) determination in orthopantomography of the angle configured between the apico-coronal axis of the included canine and the midline (alpha), and the angle determined by the former with the longitudinal axis of the definitive lateral incisor (beta) (Figure 4). In the present study, only the values of (alpha) are detailed, subdivided into two subgroups: between 0 and 60° (vertical or inclined), and above 60–100° (horizontal).

**Figure 5:**
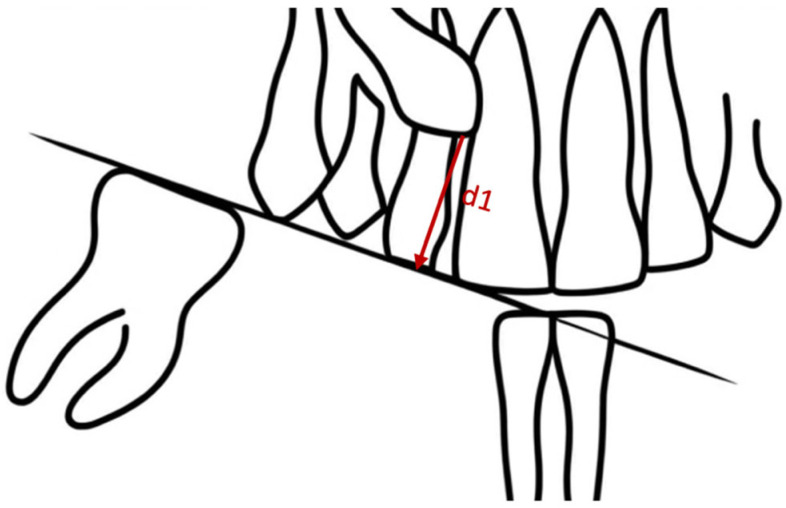
Quantification in millimeters of the height of impaction (d1).

The assessment of the height of impaction was established using a qualitative criterion [18], defining the relationship between the crown of the included canine and the apical portion, middle or coronal portion of the erupted lateral incisor (used for the present study), and a quantitative criterion, measuring the distance (d1) between the crown of the included tooth and the occlusal plane; this plane is defined as the average plane that passes through the buccal cusps of the molars and premolars and the incisal edges of the anterior teeth, serving as a guide for various dental assessments on orthopantomography (Figure 5).

**Figure 6:**
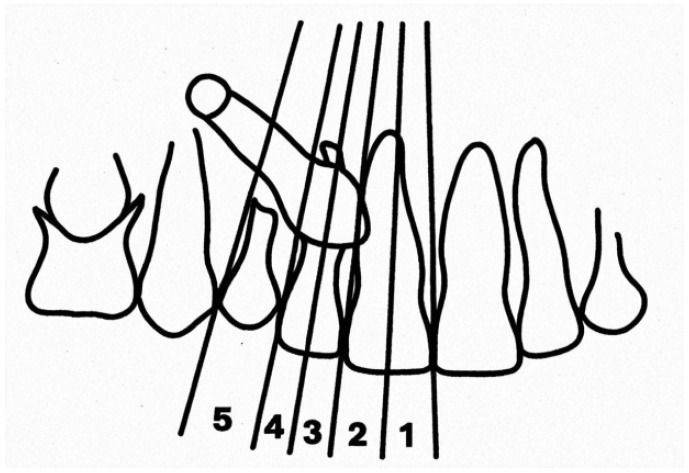
Quantification of the mesio-distal position of the impaction: 1 = canine crown more mesialized and 5 = canine crown in more distal position.

The mesio-distal position of the impaction was determined by pointing to the erupted tooth (central or lateral incisor) of which the root was closest to the crown of the included tooth, and by measuring on orthopantomography (or occlusal radiographs) the distance between the apex of the crown of the included tooth and the midline (d2), thus defining five regions in a mesio-distal direction [18]. An illustration of the five regions is displayed in Figure 6.

**Figure 7:**
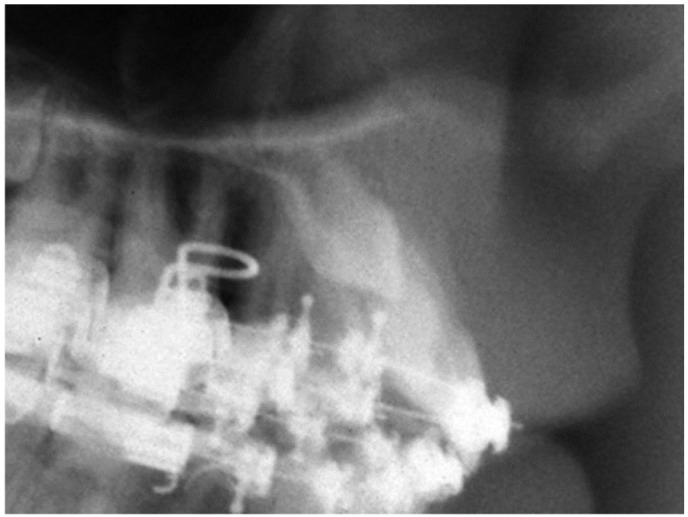
Teleradiograph: canine included by the vestibular.

Finally, the vestibular or palatal position of the included canine was determined preoperatively by periapical radiographs (Clark technique), occlusal radiograph or teleradiographs (Figure 7), confirming the radiographic information during surgery.

### 2.3. Data Analysis

Once the data were obtained, they were statistically processed using the SPSS statistical program, version 27.0.1 (SPSS Inc., Chicago, IL, USA). Descriptive statistics, including frequencies and percentages, were calculated to summarize the epidemiological information from the study. The hypothesis was tested using the chi-square test to examine relationships between categorical variables, with Fisher’s exact test employed when necessary. A *p*-value of <0.05 was considered statistically significant. The analysis was performed by two independent operators and verified by a third to ensure accuracy and minimize bias.

## 3. Results

### 3.1. Descriptive Analysis

#### 3.1.1. Age

The mean age of the patients in the sample was 21 years (range 10–41 years). By age, the group that attracted the highest number of cases was represented by patients between 14 and 30 years of age. 66.1% of the patients were female (90 women and 46 men). The female/male ratio was 1.95.

Of the total number of canines included (159 teeth), 150 (94.3%) were in the upper jaw and 9 in the mandible. The right upper canine (1.3. in the international nomenclature) was the tooth that was most frequently included in our series.

#### 3.1.2. Location of Canines (Total = 159 Canines)

Of the patients analyzed (25 patients), 18.3% had the synchronous impaction of two canines. Most of the time it was two canines in the upper jaw. Three patients had a simultaneous impaction of a canine in the upper jaw and another in the mandible. The male/female ratio in those patients with synchronous impactions (1:1) was different from that observed in patients with unitary impactions, in which the female gender clearly predominated.

#### 3.1.3. Pathology Associated with the Included Canines

**Figure 8:**
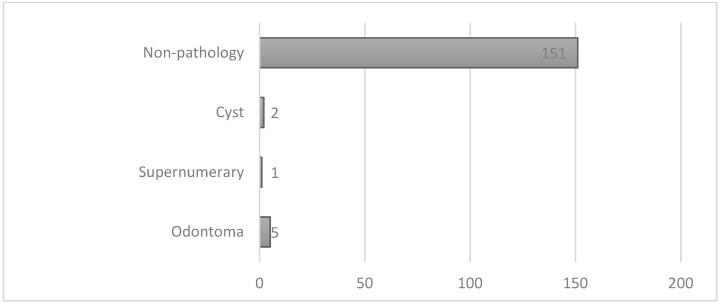
Pathologies associated with impacted canines.

Most of the patients in the series did not have an associated pathology at the time of diagnosis and treatment of their impaction. The presence of an odontoma underlying impaction was the most frequently evidenced pathology. Possible root resorption caused by the canine in neighboring teeth was not included in the present study (Figure 8).

#### 3.1.4. Proximity of the Crown of the Included Tooth to the Vestibular/Palatal Cortical and the Treatment of Your Choice

**Figure 9:**
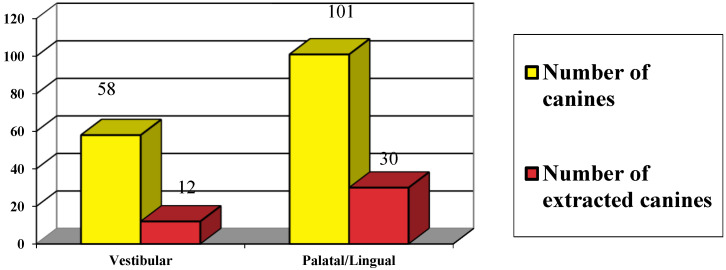
Number of canines extracted according to the proximity (vestibular or palatine) of the impacted tooth.

In relation to the palatal or vestibular location of the impacted canines, it was observed pre- and postoperatively that 62% of the teeth (101 cases) had their crowns positioned closest to the palatal plate, while 38% (58 cases) were closer to the vestibular cortical plate (Figure 9). Of those located near the vestibular plate, 20.6% (12 canines) were extracted, whereas 29.7% (30 canines) located closest to the palatal plate were exodontized. Conversely, 79.3% of the vestibular-positioned canines underwent orthodontic traction, while 70.2% of those located near the palate received the same treatment.

#### 3.1.5. Number of Canines According to the Height of the Impaction (Ratio Between the Cusp of the Included Canine and the Root of the Adjacent Lateral Incisor)

Based on the location in height of the impaction, it was found that 72% of the canines were located at the level of the middle third of the root of the lateral incisor. 24.5% of canines were positioned at the level of the apical third of the erupted lateral incisor.

Regarding the inclination of the apico-coronal axis of the canine included with the midline of the jaws, the highest percentage of cases did not present an angulation greater than 60° (97.5%), their position being defined as vertical. All canines in a horizontal position (4 cases) were extracted.

#### 3.1.6. Conservative Treatment Applied, According to Age Group

Finally, regarding the treatment applied, 75.47% of the teeth included were redirected to their location in the arch by orthodontic-surgical treatment. 24.53% of them (42 teeth) were tooth extracted.

In patients over 30 years of age, 30.43% of the included teeth were treated by orthodontic traction, while in the age group between 15–30 years this percentage rose to 85.71% (Table 1).

The inferential study of the variables in our series allows us to affirm that the type of treatment applied (extraction/traction surgery-orthodontics) has a significant relationship with the age of the patients (*p* = 0.001) (Supplementary data: Table S1). Older patients suffer more frequently from the extraction of the included canine, and this fact cannot be justified by chance.

Similarly, the greater or lesser proximity of the crown of the dental impaction to the palatal or vestibular cortical of the maxilla is significantly related to the type of treatment applied (*p* = 0.05) (Supplementary data: Table S2). In the upper jaw, the canines closest to the palatal plate are more frequently extracted than those located closer to the vestibular plate, but this fact is not justifiable by age (*p* = 0.39). We also observed that the predominant palatal location of the canines included in the upper jaw of our study (62%) has a statistically significant value (*p* = 0.002). However, we did not observe significant differences between the variable palatal/vestibular proximity of the crown of the included canine and the height (*p* = 0.5) or angulation (*p* = 0.2) of the same.

## 4. Discussion

Although the technological advances in medicine have powerfully revolutionized its scientific and practical context, epidemiological information on highly prevalent entities usually comes from classical studies. The pathology of the canines included is not alien to this situation. The work of Gupta et al. and Yang et al. constitutes the basis of epidemiological information on this pathology [2,3].

Classical studies on included canines have highlighted their relative prevalence, predominance by the female gender and the preferential proximity of their crown to the palatal/lingual plate [5,7,8,9,10,19].

The most recent studies have focused on the diagnostic reliability of conventional radiographs (periapical and orthopantomography), percentage and preoperative assessment of resorption of neighboring teeth, as well as on the analysis of the least iatrogenic surgical technique [3,6,9].

When considering imaging modalities for the evaluation of maxillary canine impactions, OPG may be preferred over CBCT for several reasons. First, OPG exposes patients to lower radiation doses compared to CBCT, making it a safer option, especially for younger patients who may be more sensitive to radiation exposure [20]. Second, OPG is generally more cost-effective than CBCT, which can be advantageous for patients seeking more affordable diagnostic options [21]. In addition, the availability of CBCT machines may be limited in public hospitals and regular dental practices, while OPG appliances are more commonly found in these settings, ensuring greater accessibility to images for the evaluation of included teeth [21]. Therefore, the use of two-dimensional diagnostic methods, such as OPG, for the evaluation of maxillary canine impactions can provide a balance between diagnostic accuracy, safety, cost-effectiveness, and accessibility, making it a suitable choice in many clinical scenarios.

In our study, we utilized two-dimensional radiographs for the diagnosis of maxillary canine impactions. It is important to acknowledge that radiographic images can be subject to various distortions, which may affect the accuracy of the measurements and interpretations. One common type of distortion is intrinsic distortion, which occurs due to the geometry of the X-ray beam and the positioning of the patient relative to the radiographic source. This can lead to discrepancies in the size and shape of the anatomical structures depicted in the image. Additionally, projection distortion can occur when the X-ray beam is not perpendicular to the area of interest, potentially resulting in magnification or foreshortening of the structures being examined. Furthermore, factors such as the patient’s head position, the angle of the X-ray beam, and the quality of the imaging equipment can contribute to variability in the resulting images. While we took measures to standardize imaging protocols and minimize these distortions, it is crucial for future studies to consider the potential impact of these factors on radiographic assessments and outcomes. In any case, distortion is present in all areas of an orthopantomography, but its value is lower in the canine region of the upper jaw [22].

The results of our study coincide with previous studies that pointed to the preferential presentation of the pathology by the female gender, but also point out that when impaction is bilateral, this difference between genders tends to disappear [2,3].

The data in our series also confirm a greater proximity of the crown of the included tooth to the palatal plate, but if a percentage close to 85% of cases is indicated in the literature for this position, in our study its value was 62%. Since the information collected on this variable in our study is a consequence of the postoperative confirmation of the dental position, we believe that it has a powerful consistency, especially when considering that the value obtained by other authors is an exclusive reflection of the radiographic assessment of impaction.

The proportion of patients with a bilateral canine impaction in our series (18.3%) is included in the range referred to in the literature on this issue (17–45%) [3]. Although we do not find a convincing explanation for this observation, it is possible that the current prevalence of unilaterally included canines has decreased in recent years, because of greater information and preventive action on forms of impaction that we could classify as “mild”. In contrast, bilateral impactions, frequently associated with marked alveolar space insufficiency, have gained greater relative weight. Authors such as Willems et al. investigated the preventive role of dentofacial orthopedics in this type of dental impactions; Some procedures such as maxillary expansion could be relevant, since the length of the arch is increased, and, consequently, orthodontic interventions can be reduced in the future [23].

At present, the objectives of orthodontic–surgical treatment of canines included in the maxilla are not only to keep the tooth in the arch, but also to do so in the best aesthetic and periodontal conditions. Therefore, multiple surgical techniques were compared, postulating that the apical replacement flap and/or complete closure of the flap after fixation with the impaction of an orthodontic anchor are the techniques with the best results [24,25].

The greater tendency for the extraction of canines included by the palatal area, although reported in the literature, has not been statistically investigated. Some authors point out that the greater thickness of the cortical and palatal mucosa would justify this fact, but the most accepted cause for the moment is the lack of space in the arch, which is much more common in impactions, among others [25].

Our study highlights that the extraction of the included canines is significantly conditioned by the age of the patient to the treatment, as well as by their greater proximity to the palatal plate. However, the latter fact appears to be independent of age.

The results of our study show no significant differences between the position (vestibular/palatal) of the impaction and its height or angulation.

It would be logical to suppose that the greater tendency to extraction of the canines included by the palatal was influenced by a more unfavorable spatial arrangement inside the maxilla. Canines included by the palatal would be at a higher height or with a greater angulation than those included by the vestibule, and for this reason there would be a natural tendency towards their extraction.

The present study has several limitations that warrant consideration. Firstly, the sample size was relatively small, which may affect the statistical power and generalizability of the findings. Additionally, the study was conducted in a specific geographic region, focusing solely on patients from Asturias, Spain. This limitation restricts the diversity of the sample and may not accurately represent the broader population. To enhance the robustness and applicability of the results, a multicentric study design is recommended. Such a study should include participants from multiple countries, various hospitals, and clinics, thereby facilitating the inclusion of a larger and more heterogeneous patient population. This approach would provide a more comprehensive understanding of maxillary canine impactions and their management across different demographics and clinical settings. The retrospective design of this study entails that data collection occurred following the clinical decision-making process, which restricts any possibility of altering treatment protocols. This limitation introduces potential variability in the outcomes, a factor we have duly acknowledged in the discussion section. To mitigate this limitation and strengthen the validity of future findings, we propose conducting a prospective observational study, allowing for greater control over variables and providing a more robust basis for comparison and validation of the results.

The clinical relevance of this study lies in its potential to enhance the diagnostic approach for impacted canines using conventional radiographs instead of more advanced imaging techniques like CBCT. By focusing on radiographic analysis, we can achieve accurate diagnosis and treatment planning while minimizing the radiation exposure for patients. This study aimed to establish reliable criteria that can guide clinicians in identifying impacted canines effectively, leading to timely interventions and improved patient outcomes. Additionally, the findings may inform orthodontic treatment decisions, reducing the need for invasive procedures and promoting more conservative management strategies. Ultimately, this research contributes to optimizing patient care and resource utilization in dental practices.

## 5. Conclusions

The diagnosis and treatment of impacted canines in our environment predominantly occur in individuals aged 14 to 30 years, with a higher prevalence in women. Canines impacted on the palatal side, as well as those diagnosed in older patients, show a significantly greater likelihood of requiring extraction. However, when comparing palatally impacted canines to those impacted on the vestibular side, no significant differences were observed in terms of height or angulation, suggesting similar clinical presentations despite differing impaction locations.

## Figures and Tables

**Table 1:** Treatment applied according to age in our series.

Age Range	Total Teeth	Tractioned Teeth (75.47%)	Teeth Extracted (24.53%)
Less than 15 years	19.5% (31)	74.19% (23)	25.81% (8)
15 to 30 years old	66.04% (105)	85.71% (90)	14.29% (15)
More than 30 years	14.47% (23)	30.43% (7)	69.57% (16)

## Data Availability

The original contributions presented in the study are included in the article.

## Supplementary Materials

The following supporting information can be downloaded at: https://www.mdpi.com/article/10.3390/dj12110360/s1, Table S1: Age and type of treatment applied; Table S2: Cortical and type of treatment applied.
