# Supplementary material for: Two-Dimensional Radiographic Diagnosis of Maxillary Canine Impactions
Source: Dent J (Basel). 2024 Nov 13;12(11):360. doi: 10.3390/dj12110360 (PMC11593121; doi:10.3390/dj12110360)
Supplement: Supplementary file 1 [file dentistry-12-00360-s001.zip › dentistry-3246791-supplementary.pdf]

Supplementary Materials

Table S1. AGE AND TYPE OF TREATMENT APPLIED

		Extraction	Traction	Total
AGE	Adult	17	7	24
	Young	20	84	104
	Child	8	23	31
Total		45	114	159

Chi-Square Tests

	Value	df	Asymp. Sig. (2-sided)
Pearson Chi-Square	28,884	4	0.000
Likelihood Ratio	27,026	4	0.000
N of Valid Cases	159		

Table S2. CORTICAL AND TYPE OF TREATMENT APPLIED

		Extraction	Traction	Total
CORTICAL	Palatal	33	67	100
	Vestibular	12	47	59
Total		45	114	159

Chi-Square Tests

	Value	df	Asymp. Sig. (2-sided)
Pearson Chi-Square	5,609	2	0.061
Likelihood Ratio	5,834	2	0.050
N of Valid Cases	159		
